# Dentin erosive wear is reduced by fluoride varnishes containing nanosized sodium trimetaphosphate *in vitro*

**DOI:** 10.1590/1807-3107bor-2024.vol38.0056

**Published:** 2024-07-15

**Authors:** Isabela Ferreira da SILVA, Letícia Cabrera CAPALBO, Renan DAL-FABBRO, Mayra Frasson PAIVA, Thayse Yumi HOSIDA, Liliana Carolina BÁEZ-QUINTERO, Caio SAMPAIO, Douglas Roberto MONTEIRO, Alberto Carlos Botazzo DELBEM, Juliano Pelim PESSAN

**Affiliations:** (a) Universidade Estadual Paulista – Unesp, School of Dentistry of Araçatuba, Department of Preventive and Restorative Dentistry, Araçatuba, SP, Brazil.; (b) University of Michigan, School of Dentistry, Department of Cariology, Restorative Sciences, and Endodontics, Ann Arbor, MI, USA.; (c) Universidad Cooperativa de Colombia, Facultad de Odontologia, Bogotá, Colombia.

**Keywords:** Phosphates, Fluorides, Tooth Erosion, Dentin

## Abstract

This study evaluated the effect of fluoride varnishes containing micrometric or nanosized sodium trimetaphosphate (TMP) on dentin erosive wear *in vitro*. Bovine root dentin blocks were selected by surface hardness and randomly divided into five experimental groups/varnishes (n = 20/group): placebo, 5% sodium fluoride (NaF); 5% NaF+5% micrometric TMP; 5% NaF+2.5% nanosized TMP; and 5% NaF+5% nanosized TMP. Half of the surface of all blocks received a single application of the assigned varnish, with subsequent immersion in artificial saliva for 6 h. Varnishes were then removed and the blocks were immersed in citric acid (90 s, 4×/day, 5 days). After each erosive cycle, ten blocks of each group were immersed in a placebo dentifrice for 15 s (ERO), while the other ten blocks were subjected to abrasion by brushing (ERO+ABR). Dentin erosive wear was assessed by profilometry. Data were submitted to 2-way ANOVA and to the Holm-Sidak test (p<0.05). Dentin erosive wear was significantly higher for ERO+ABR than for ERO for all varnishes. TMP-containing varnishes promoted superior effects against dentin erosive wear compared with 5% NaF alone; and 5% nanosized TMP led to the lowest wear among all varnishes. In conclusion, the addition of TMP to conventional fluoride varnish (*i.e.*, varnish containing only NaF) enhanced its protective effects against bovine root dentin erosion and erosion+abrasion. Additionally, the use of 5% nanosized TMP led to superior effects in comparison to 5% micrometric TMP, both for erosion and erosion+abrasion *in vitro*.

## Introduction

Dental erosion is an increasing dental condition worldwide, affecting 30% to 50% of deciduous teeth and 20% to 45% of permanent teeth.^1^ The association of the exposure to intrinsic or extrinsic acids (not derived from bacteria) and mechanical impacts (*i.e.*, abrasion) leads to a cumulative loss of the tooth surface, named erosive tooth wear (ETW).^2-4^ Therapies targeted at this condition seek to prevent and/or reduce the progression of erosive lesions, and preventive strategies involve changes in eating habits (reducing the intake of acidic foods and beverages) and application of topical fluoridated products.^5-7^ Even though ETW is mostly studied on enamel, it can also affect the dentin tissue which, when untreated, may cause discomfort to the patient, leading to dentin hypersensitivity, especially when consuming cold foods and/or beverages.

Although the clinical effectiveness of fluoridated products against the onset and progression of carious lesions is well established, these products have a limited effect on ETW.^8^ This occurs due to the fast dissolution of the hydroxyapatite crystals from the surface layer after erosive challenges, which reduces fluoride effects on the rehardening/remineralizing processes.^9^ Furthermore, on the dentin tissue, the effects of fluoride are more evident and effective when used at high concentrations.^10^ Within this context, fluoride varnishes stand out, considering their wide use in clinical practice because of their easy application, safety, and efficacy.^11^

Varnishes are viscous products in which fluoride (usually at high concentrations) is embedded in a polymeric base, which forms a thin film on the tooth surfaces that releases fluoride over prolonged periods.^12^ The most common fluoride-containing varnishes use colophony (a natural polymer) in their composition. In order to enhance the effects of fluoridated varnishes, the addition of phosphates, such as sodium trimetaphosphate (TMP), to conventional fluoride varnishes was shown to promote a higher reduction of enamel wear.^12^ In addition, the use of TMP nanoparticles was shown to further increase the protective effect of these varnishes on ETW when compared with microparticles.^13^ However, the effects of fluoride varnishes containing nanosized TMP on enamel (which only contains apatite crystals) cannot be directly extrapolated to dentin (which comprises both organic and inorganic content), given that TMP’s action can be affected by its interaction with the dentin organic matrix.^14^ This points out to the need for studies on ETW using tooth dentin as a substrate.

Accordingly, this study aimed to evaluate the protective effects of micrometric or nanosized TMP against bovine root dentin erosive wear using erosion or erosion-abrasion cycling models. The null hypotheses of the study were that (1) the incorporation of TMP into fluoride varnishes would not enhance their protective effects, and (2) the use of TMP nanoparticles would not lead to superior protective effects over microparticles.

## Methods

### Products

The same varnishes used by Paiva et al.^13^ were used in the present study. The products were manufactured by SS White Produtos Odontológicos (Rio de Janeiro, RJ, Brazil) and consisted of a film-forming polymer (artificial resin), solvent (ethanol), essence (saccharin), and demineralized water (q.s.p.). For the varnishes containing nanosized TMP, the nanoparticles were synthesized and characterized at the Federal University of São Carlos (UFSCar) using commercial TMP (Na_3_O_9_P_3_, Sigma-Aldrich, purity ≥ 95% CAS 7785-84-4). These nanoparticles exhibited an average particle size of 22.7 nm while maintaining their crystalline structure without any alterations.^14^ The varnishes were formulated to contain 5% NaF, with the option of including 5% micrometric TMP or 2.5% or 5% nanosized TMP. Moreover, a placebo varnish (negative control) without the addition of NaF or TMP was prepared.^13^

Prior to the experiments, the F concentrations of the varnishes were determined using an ion-specific electrode (9609 BN - Orion) and ion analyzer (Orion 720 A+), which were calibrated with standards ranging from 2.0 to 32.0 μg F/mL, following the protocol described by Manarelli et al.^10^ The mean (standard deviations) F concentrations of the varnishes were as follows: Placebo - 433.6 (33.5) μg F/g; 5% NaF - 21,378.8 (708.1) μg F/g; 5% NaF + 5% micrometric TMP - 20,154.0 (326.9) μg F/g; 5% NaF + 2.5% nanosized TMP - 20,063.5 (301.7) μg F/g; and 5% NaF + 5% nanosized TMP - 20,154.0 (326.9) μg F/g, as described by Paiva et al.^13^

### Blocks preparation

Dentin blocks (n = 100, 4 × 4 × 2 mm), obtained from bovine permanent mandibular central incisor roots were cut and sequentially polished with 320-, 600-, 800-, and 1200-grit grinder polishers (CARBIMET PaperDiscs, 30-5108-320, BUEHLER), with a final polish using a felt disc (Buehler Polishing Cloth 40–7618) moistened with a 1-μm diamond polishing suspension (Extec Corp., Enfield,CT, USA).^15^ The number of blocks used in the study was determined using data from a pilot study carried out with four dentin blocks per group, according to which seven blocks would be required to detect significant differences between groups treated with 5% microparticles and 5% nanoparticles for erosive challenge, considering a mean difference of 1.3, a standard deviation of 0.6, and power of 80% (α=0.05). Ten blocks (including those used in the pilot study) were included in each group to make up for possible losses during treatment, cycling, and analysis.

### Experimental design

Bovine dentin blocks (n = 100) were selected according to their surface hardness after surface hardness analysis (SH), using the Micromet 5114 hardness tester (Buehler, Lake Bluff, USA and Mitutoyo Corporation, Kanagawa, Japan). This study followed a 5 × 2 factorial design, considering (a) treatments at five levels (five varnishes) and (b) cycling models at two levels (erosion or erosion+abrasion) as experimental factors. The blocks were randomly assigned to five experimental groups according to the varnishes tested (n = 20/group): Placebo (Pla - without F or TMP); 5% NaF (5% NaF); 5% NaF + 5% micrometric TMP (5% microparticles); 5% NaF + 2.5% nanosized TMP (2.5% nanoparticles); and 5% NaF + 5% nanosized TMP (5% nanoparticles), following a single-blinded protocol.

Half of the surface of each block was protected with acid-resistant nail varnish (control area) prior to receiving a single application of the varnishes. A thin layer of the varnishes was applied in the test area of the dentin blocks, which were then immersed in artificial saliva (1.5 mmol.l^1^ Ca(NO_3_)_2_∙4H_2_O; 0.9 mmol. l^1^ NaH_2_PO_4_∙2H_2_O; 150 mmol.l^1^ KCl; 0.1 mol.l^1^; Tris buffer) for 6 h. The varnishes were then removed with a spatula and acetone (Sigma-Aldrich CO., St. Louis, USA), and the blocks were washed with deionized water and dried with absorbent paper. All blocks were subjected to four daily erosion challenges for 5 days (ERO, immersion in 0.05 M citric acid (Synth®, Brazil), pH 3.2, 90 s/cycle, stirring 35/min). After each erosive challenge, half of the blocks (n=10/group) were immersed in a slurry of placebo dentifrice for 15 s, under stirring at 35/min.^16^ The other half of the samples were subjected to abrasion (ERO+ABR) challenge using a brushing machine (axial load 250 g, five back-and-forth movements/s, 15 s; Elquip Maq Escovação, São Carlos, Brazil) and a slurry of the same placebo dentifrice. For both the ERO and ERO+ABR, , the blocks remained for 2 h in artificial saliva (pH 7.0; no agitation) between each erosive challenge (Figure).

**Figure:**
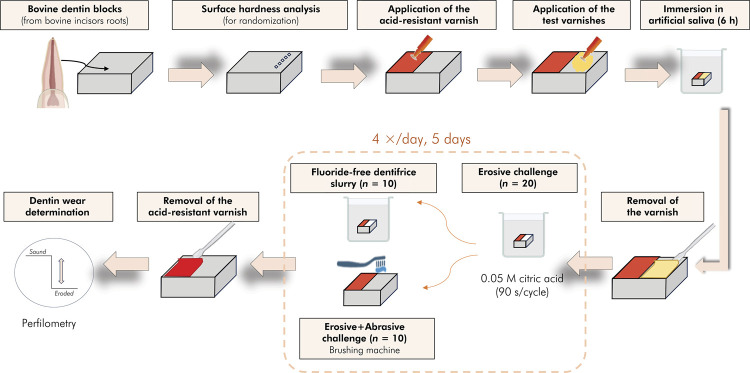
Flowchart summarizing the study design.

### Determination of surface wear

For the profilometric analysis, the acid-resistant nail polish was carefully removed from the control surface with a spatula and a cotton swab, soaked in acetone. Dentin wear was determined by profilometric analysis, in which the tip of the roughness tester (Surftest SJ 401, Mitutoyo American Corporation, Kanagawa, Japan) ran along the sound surface of the specimens towards the eroded area. Five readings were taken 0.5 mm apart at the center of the sample to calculate the average wear for each block.^9,17^

### Statistical analysis

Data did not pass normality (Shapiro-Wilk) and homogeneity (Barlett) tests (even after cubic or square root transformations), and were subjected to 2-way ANOVA, followed by the Holm-Sidak test. The SigmaPlot software, version 12.0, was used, and the significance level was set at 5%.

## Results

Significant differences were observed between the challenges (F = 150.1, p < 0.001) and among the treatments (F = 432.3, p < 0.001), with no significant interactions between challenges and treatments (F = 0.9, p = 0.475). Bovine root dentin wear was significantly higher for ERO + ABR than for ERO for all varnishes. Furthermore, significant differences in bovine root dentin wear were observed among all varnishes under both conditions (ERO or ERO + ABR), except between 2.5% nanoparticles and 5% microparticles. Also, the addition of TMP, regardless of the particle size or concentration, enhanced the protective effect of the 5% NaF varnish for both the ERO and ERO + ABR conditions. The use of 5% nanovarnish led to the lowest bovine root dentin wear values among all study groups (Table).

**Table:** Mean (SD) values of dentin wear (µm) after treatment with the varnishes and exposure to erosive (ERO) or erosive+abrasive (ERO+ABR) challenges.

Challenges	Varnishes				
	Pla	5% NaF	5% micro	2.5% nano	5% nano
ERO	16.8^A,a^	7.1^A,b^	5.8^A,c^	5.9^A,c^	4.6^A,d^
	(1.0)	(0.6)	(0.4)	(0.7)	(0.5)
ERO+ABR	20.3^B,a^	9.6^B,b^	8.6^B,c^	8.7^B,c^	6.9^B,d^
	(1.8)	(1.8)	(1.4)	(1.0)	(0.9)

Different uppercase letters indicate significant differences between ERO and ERO+ABR within each varnish. Different lowercase letters indicate significant differences among the varnishes within each challenge. Two-way ANOVA and the Holm-Sidak test (p < 0.05, n = 10/group for each challenge). Pla = placebo (without any active compound); 5% NaF = 5% sodium fluoride; 5% microparticles = 5% NaF + 5% micrometric TMP; 2.5% nanoparticles = 5% NaF + 2.5% nanosized TMP; 5% nanoparticles = 5% NaF + 5% nanosized TMP.

## Discussion

ETW can negatively affect the quality of life of patients in advanced stages^18,19^ because of its impact on aesthetics along with functional damage^20^ and pain (hypersensitivity).^21^ Considering the cumulative effect of ETW throughout life, strategies to prevent this condition are paramount, although dentists around the world still struggle to find effective, affordable, and minimally invasive measures to prevent or reduce ETW. The present study evaluated the supplementation of fluoridated varnishes with TMP and, to the authors’ knowledge, this is the first investigation assessing the protective effects of nanosized TMP against erosive wear in bovine root dentin. Our results showed that the supplementation of fluoridated varnishes with TMP, regardless of the particle size, was more effective in reducing dentin erosive wear compared to a conventional formulation containing only 5% NaF as active compound and, therefore, the first null hypothesis was rejected. Additionally, the use of TMP nanoparticles proved to have a higher protective effect than the TMP microparticles at a comparable concentration (5%), thus leading to the rejection of the second null hypothesis.

The addition of phosphates to fluoridated products has been shown to be an effective alternative for the protection against erosive wear on dental enamel.^12,22^ Nonetheless, the erosive process can also affect the underlying dentin structure, as occurs in erosive lesions at more advanced stages and in lesions in the cervical region. In this sense, structural differences between enamel and dentin regarding their mineral composition, structural arrangement, and protein content make each of these dental tissues respond differently to the same treatment when exposed to erosive and abrasive challenges; therefore, evidence for enamel cannot be directly extrapolated to dentin.

The aforementioned rationale is supported by the comparison of the data from the present study (dentin erosion) with those from a previous work,^13^ both conducted by the same research group, using the same varnishes and following an identical protocol for erosive and abrasive challenges. While the overall bovine enamel erosive wear for ERO+ABR was ~20% higher than the one observed for blocks only subjected to ERO,^13^ in the present study, the overall dentin erosive wear was 40% higher for ERO+ABR than for ERO. These findings are in line with those from previous studies reporting a higher impact of abrasion on dentin erosive wear^24,25^ compared with enamel.^23^ This trend, however, should be interpreted with caution, as the aforementioned studies were performed on different occasions and by different investigators, making direct comparisons somewhat inappropriate.

Interestingly, on the other hand, dentin was shown to be more responsive than enamel for all treatments, both for ERO and ERO+ABR. For example, for dentin ERO (present study), the use of 5% NaF, 5% microparticles and 5% nanoparticles reduced ETW by 55.9%, 64.0%, and 71.4%, respectively, in comparison to Pla, while the corresponding values for enamel ERO^13^ were 37.7%, 46.2%, and 60.4%. The lower mineral content of dentin (~70%) compared with enamel (~96%) and the presence of dentinal tubules (which increase the surface area and allow for a deeper penetration of the citric acid into the tubules) may be regarded as the main cause for the higher susceptibility of dentin to tissue loss compared with enamel.

Also, a better performance of nanoparticles over micrometric ones was observed against ETW both for enamel^13^ and dentin (present study). In fact, in both studies, the use of nanoparticles led to a similar additional effect over microparticles, considering that the 5% nanovarnish promoted a protective effect ~20% higher than the 5% microparticles, under both experimental conditions (ERO and ERO+ABR). In that regard, it is important to highlight that the superior effect of TMP nanoparticles over microparticles was virtually the same for dentin under ERO (20.7%) or ERO+ABR (19.8%), while for enamel, the corresponding values were 26.3% and 13.8%.^13^ Despite not investigated in the present study, the trends described above might also be explained by structural differences between enamel and dentin, especially regarding the surface area for the binding of active compounds, as described below.

For enamel, the entire surface is exposed to erosive challenges and to abrasion by brushing, so that the protective layer formed by the active compounds may be slowly removed over time, which is why the formation of an acid-resistant barrier can be as advantageous as promoting effects on enamel rehardening, given that repetitive erosive challenges may hinder mineral precipitation.^13,26,27^ For dentin, on the other hand, its complex three-dimensional structure allows for the binding of the active compounds both on the external surface (*i.e.*, intertubular dentin) and on the walls of the tubules (*i.e.*, peritubular dentin), so that the abrasive challenges will mostly affect the protective layer on the external surface. Furthermore, in addition to the mineralizing effects of fluoride and TMP, both active compounds have antiproteolytic potential,^14^ which can be directly related to their more evident performance in dentin, considering that both active compounds not only protect the inorganic compound of the tissue, but also the organic compounds.

Interestingly, no significant differences were observed between the groups treated with varnishes supplemented with 5% TMP microparticles and 2.5% TMP nanoparticles. In fact, this pattern has also been previously observed for enamel demineralization in a study showing that a dentifrice supplemented with TMP nanoparticulated at 1% promoted the same protective effect compared to TMP microparticulated at 3%.^28^ This reinforces the concept that nanoparticles allow for the use of lower concentrations of the phosphate without reducing its preventive/therapeutic effects. Furthermore, the use of TMP nanoparticles at the same concentration of TMP microparticles resulted in a higher protective effect of nanoparticles over micrometric ones, which has also been observed in the study by Danelon et al.^25^ The better performance of nanosized over micrometric particles can be explained by the greater surface contact of nanoparticles, in addition to the high percentage of atoms on their surface compared to microparticles.^29-31^ Such properties of the nanoparticles seem to potentiate the effects of the phosphate itself, which has been regarded as a barrier against acid diffusion, which also allows for the retention of ions and species that take part in remineralization processes.^10,12^

Despite the trends described above, the results found must be interpreted with caution given certain limitations imposed by the study protocol. Firstly, in order to promote highly controlled conditions and to determine the isolated effect of the varnishes tested, this protocol did not consider the simultaneous effects of varnishes (professional application) with the routine use of fluoridated toothpaste (self-application at home). Thus, future studies comprising this variable (*i.e.*, the use of fluoridated toothpaste) should be carried out in order to better mimic more realistic conditions. It is also noteworthy that the use of an *in vitro* protocol and artificial saliva prevents the results from being directly extrapolated to complex reality, given the oral dynamics found in vivo. Lastly, it is crucial to note that bovine dentin samples were utilized in this study. Although this tissue has been widely utilized due to its good reliability for this purpose,^14,15^ it is important to acknowledge that human teeth may exhibit distinct patterns.

Notwithstanding the aforementioned limitations, the results obtained are promising and have great potential for clinical applicability, especially considering the current results in light of the cumulative and irreversible effects of dentin erosive wear. Thus, further studies comprising protocols closer to clinical conditions (*i.e., in situ* and clinical studies) that properly address the aforementioned limitations and that may provide additional information to assess the real benefit of supplementation with nanosized TMP should be carried out.

## Conclusions

Based on the results presented herein, in can be concluded that the addition of TMP to a conventional fluoride varnish (i.e., varnish containing only NaF) enhanced its protective effects against bovine root dentin erosion and erosion+abrasion. Additionally, the use of 5% nanosized TMP led to superior effects in comparison to 5% micrometric TMP, both for erosion and erosion+abrasion *in vitro*.
